# Revealing complexity: beyond the whole—segmentation of hippocampal subfields in adolescents with depression and its relationships with cognition

**DOI:** 10.1192/j.eurpsy.2024.481

**Published:** 2024-08-27

**Authors:** Y. Zhang, X. Liu, Y. Yang, F. Xu, D. Yu, X. Zhu, K. Wang, W. Zhang

**Affiliations:** ^1^School of Psychology, Shandong Normal University; ^2^Shandong Mental Health Center; ^3^Radiology Department of Qilu Hospital, Shandong University, Jinan, China; ^4^Institute of Health and Wellbeing, University of Glasgow, Glasgow, United Kingdom

## Abstract

**Introduction:**

The occurrence of depression in adolescence, a critical period of brain development, linked with neuroanatomical and cognitive abnormalities. Neuroimaging studies have identified hippocampal abnormalities in those of adolescent patients. However, few studies have investigated the atypically developmental trends in hippocampal subfields in adolescents with depression and their relationships with cognitive dysfunctions.

**Objectives:**

To explore the structural abnormalities of hippocampal subfields in patients with youth depression and examine how these abnormalities associated with cognitive deficits.

**Methods:**

We included a sample of 79 first-episode depressive patients (17 males, age = 15.54±1.83) and 71 healthy controls (23 males, age = 16.18±2.85). The severity of these adolescent patients was assessed by depression scale, suicidal risk and self-harm behavior. Nine cognitive tasks were used to evaluate memory, cognitive control and attention abilities for all participants. Bilateral hippocampus were segmented into 12 subfields with T1 and T2 weighted images using Freesurfer v6.0. A mixed analysis of variance was performed to assess the differences in subfields volumes between all patients and controls, and between patients with mild and severe depression. Finally, LASSO regression was conducted to explore the associations between hippocampal subfields and cognitive abnormalities in patients.

**Results:**

We found significant subfields atrophy in the CA1, CA2/3, CA4, dentate gyrus, hippocampal fissure, hippocampal tail and molecular layer subfields in patients. For those patients with severe depression, hippocampal subfields showed greater extensive atrophy than those in mild, particularly in CA1-4 subfields extending towards the subiculum. These results were similar across various severity assessments. Regression indicated that hippocampal subfields abnormalities had the strongest associations with memory dysfunction, and relatively week associations with cognitive control and attention. Notably, CA4 and dentate gyrus had the highest weights in the regression model.

**Conclusions:**

As depressive severity increases, hippocampal subfield atrophy tends to spread from CA regions to surrounding areas, and primarily affects memory function in patients with youth depression. These results suggest hippocampus might be markers in progression of adolescent depression, offering new directions for early clinical intervention.

**Disclosure of Interest:**

None Declared

